# Clinical presentation and outcome of Graves disease in pediatric patients with and without type 1 diabetes: a retrospective cohort study

**DOI:** 10.3389/fendo.2025.1588587

**Published:** 2025-06-27

**Authors:** Yi-Hsin Wu, Lu-Ting Wang, Yann-Jinn Lee, Chi-Yu Huang, Yu-En Kao, Chao-Hsu Lin, Bi-Wen Cheng, Fu-Sung Lo, Wei-Hsin Ting

**Affiliations:** ^1^ Department of Pediatrics, Shin-Kong Wu Ho-Su Memorial Hospital, Taipei, Taiwan; ^2^ Department of Pediatric Endocrinology, MacKay Children’s Hospital, Taipei, Taiwan; ^3^ Department of Medicine, MacKay Medical College, New Taipei, Taiwan; ^4^ Department of Medical Research, Tamsui MacKay Memorial Hospital, New Taipei, Taiwan; ^5^ Institute of Biomedical Sciences, MacKay Medical College, New Taipei, Taiwan; ^6^ Department of Pediatrics, School of Medicine, College of Medicine, Taipei Medical University, Taipei, Taiwan; ^7^ Department of Pediatric Endocrinology, Hsinchu Municipal Mackay Children’s Hospital, Hsinchu, Taiwan; ^8^ Department of Biological Science and Technology, National Yang Ming Chiao Tung University, Hsinchu, Taiwan; ^9^ Department of Pediatrics, Chang Gung Memorial Hospital, Taoyuan, Taiwan; ^10^ College of Medicine, Chang Gung University, Taoyuan, Taiwan

**Keywords:** autoimmune thyroid disease, thyrotoxicosis, Graves disease, type 1 diabetes, pediatric, remission

## Abstract

**Introduction:**

Type 1 diabetes (T1D) is associated with autoimmune thyroid disease (AITD). However, few studies have explored the clinical presentation and outcomes of Graves disease (GD) in T1D patients. In this study, we examined the clinical manifestation and compared the remission rates of GD in pediatric patients with and without T1D.

**Materials and Methods:**

Patients with T1D diagnosed at ≤ 18 years at MacKay Children’s Hospital, Taipei, from 2000 to 2020 underwent annual screening for AITD. Those who met the criteria for GD were enrolled in the study. Clinical manifestations, demographic data, thyroid function test results, methimazole dosage and outcome data were collected and compared with those pediatric GD patients without T1D.

**Results:**

Of the 651 pediatric patients with T1D, 15 (2.3%) developed GD either with or after their T1D diagnosis. At the time of GD diagnosis, 11 of 15 GD patients with T1D (73.3%), and 98.9% patients without T1D exhibited symptoms of thyrotoxicosis, indicating a significant difference (*p* < 0.001). GD patients with T1D had significantly lower median free T4 levels (2.2 vs. 4.1 ng/dL, *p* < 0.001) and TSH receptor antibody (TRAb) (27.2% vs. 58.5%, *p* = 0.004) compared with those patients without T1D.

Following treatment, free T4 levels normalized more rapidly in GD with T1D group (2.2 vs. 3.5 months, *p* = 0.031) with a lower dosage of methimazole (0.1 vs. 0.33 mg/kg/day, *p* < 0.001). However, no significant difference was observed in remission rate (33.3% vs. 23.4%, *p* = 0.377) or time to remission (3.3 vs. 4.5 years, *p* = 0.446) between the two groups.

**Conclusion:**

The prevalence of GD in children and adolescents with T1D was 2.3%. Compared with GD patients without T1D, those with T1D exhibited fewer thyrotoxic symptoms and had lower free T4 and TRAb levels at the time of GD diagnosis. Although the remission rate did not significantly differ between the two groups, annual screening for GD should still be considered because it assisted in the rapid relief of thyrotoxicosis with relatively low dosage of antithyroid drugs.

## Introduction

It is well known that patients with type 1 diabetes (T1D) have an increased predisposition to developing additional autoimmune diseases (1–4). The most common condition is autoimmune thyroid disease (AITD), with Hashimoto’s disease being considerably more prevalent than Graves disease (GD) (2–6). The prevalence of GD in Caucasian and Australian children with T1D has been reported to range from 0.27% to 3% (7–11). However, few studies have examined the prevalence of GD in Asian children and adolescents with T1D.

Despite the low prevalence of GD in patients with T1D, hyperthyroidism has a major effect on glucose metabolism in this population. It stimulates gluconeogenesis and glycogenolysis, reduces insulin sensitivity, and increases glucose uptake and lipolysis in muscles, leading to the deterioration of metabolic control (12). Several studies have indicated that thyrotoxicosis can lead to diabetic ketoacidosis in patients with T1D (13, 14). Dost et al. (9) reported that hyperthyroidism in children with T1D is associated with an increased risk of ketoacidosis and hypoglycemia compared with euthyroid T1D patients. Despite these findings, few studies have explored the clinical presentation and evolution of GD in patients with T1D.

In this study, we examined the prevalence of GD in children and adolescents with T1D. We also described and compared the clinical presentations, laboratory findings, antithyroid drug dosages, and remission rates of GD in children and adolescents with and without T1D.

## Materials and methods

Children and adolescents diagnosed with T1D at ≤18 years of age and were regularly followed up at MacKay Children’s Hospital between 2000 and 2020 received annual screening procedures for AITD. These procedures included measurements of free T4, thyroid-stimulating hormone (TSH), and thyroid autoantibodies, such as thyroid peroxidase antibody, thyroglobulin antibody, and TSH receptor antibody (TRAb). Patients meeting the following criteria for pediatric GD were included in the study: (1) having high thyroid hormone levels (free or total T4) with suppressed serum TSH levels, (2) testing positive for TRAb, and (3) having GD diagnosed at ≤18 years of age. During the same period, patients with GD (diagnosed before 18 years of age) without T1D were enrolled as control group. Patients with follow-up durations of less than 1 year and those who had any chromosomal anomalies (e.g., Down or Turner syndrome) were excluded from the analysis.

Data on the patients’ sex, age at GD and T1D diagnosis, body mass index (BMI) *z*-score, pubertal status, family history of thyroid disease (e.g., AITD, goiter, thyroid nodules, or thyroid cancer), thyrotoxic symptoms (e.g., tachycardia, shortness of breath, body weight loss, polyphagia, exophthalmos, palpitation, goiter, or tremor), thyroid function test results, TRAb levels at GD diagnosis, initial antithyroid drug (methimazole) dosages, and side effect of methimazole were collected from their electronic medical records. Remission was defined as the maintenance of euthyroid for at least 12 months after the discontinuation of antithyroid drug (ATD) therapy without relapse during the study period. Relapse was defined as elevated free T4 with suppressed TSH levels after discontinuation of ATD and warranted reuse of ATD. Definitive therapy, including surgery or radioiodine ablation (RAI) was administered in cases of relapse or prolonged methimazole for more than 2 years.

Free T4 was measured by radioimmunoassay (Beckman Coulter, Prague, Czechia; reference range: 0.89–1.79 ng/dL), TSH was measured by radioimmunometric assay (Cisbio, Codolet, France; reference range: 0.25–4.00 μIU/mL), and TRAb was measured by radioreceptor assay (RRA) kit (Cisbio, Cardiff, UK; reference range: <15%).

The study was approved by the Institutional Review Board of MacKay Memorial Hospital, Taipei, Taiwan. And the patients and/or their guardians gave informed consent.

### Statistical analysis

Categorical variables are presented as frequencies and percentages, and continuous variables are presented as medians with interquartile ranges (25th to 75th percentile). BMI is reported as a standard deviation score compared with that of an age- and sex-matched reference population (15). Between-group comparisons were conducted using the Mann–Whitney *U* test for nonparametric continuous variables and using chi-square or Fisher’s exact tests in cases of fewer than five expected frequencies for categorical variables. We performed a linear mixed model analysis with pairwise comparison to examine the changes in TRAb levels over time between patients with and without T1D. All statistical analyses were conducted using SPSS V. 26 (IBM, Armonk, NY, USA), with a *p* value of < 0.05 considered statistically significant.

## Results

### Baseline characteristics of GD patients with and without T1D

Of 651 patients with T1D, 15 (2.3%) developed GD before the age of 18 years. Among them, 10 were girls and 5 were boys. The median age at T1D diagnosis was 7.7 years (interquartile range (IQR): 5–13.1 years), and the median age at GD diagnosis was 11.3 years (IQR: 8.5–16.1 years), with a median diabetes duration of 3.1 years (IQR: 0–4.2 years).

A total of 348 patients met the criteria for pediatric GD without T1D. Of these patients, 57 were excluded because they either had a short follow-up period (less than 1 year, *n* = 45) or had any chromosomal anomalies (*n* = 12), leaving 291 patients enrolled in the control group.

**Table 1** presents a summary of the clinical characteristics and laboratory findings of pediatric GD patients with and without T1D. No significant differences were observed between the two groups in terms of age at GD diagnosis, BMI *z*-score, pubertal status, or family history of thyroid disease. However, the proportion of girls was significantly lower in GD with T1D group compared to those without T1D (10/15 vs. 86.9%, *p* = 0.028).

**Table 1:** Clinical and laboratory characteristics of pediatric GD patients with and without T1D.

Variable	N	GD with T1D N=15	GD without T1D N=291	*P* value
Female, n/total count (%)	306	10/15 (66.7%)	253/291 (86.9%)	0.03
Age at T1D diagnosis (years)	15	7.7 (5-13.1)	N/A	
Duration from T1D to GD (years)	15	3.1 (0-4.2)	N/A	
Age at GD diagnosis (years)	306	11.3 (8.5-16.1)	12.3 (10-5.3)	0.47
BMI (z-score)	294	-0.5 ± 0.6	-0.4 ± 0.9	0.57
Pubertal, n/total count (%)	285	8/12 (66.7%)	204/273 (74.7%)	0.53
Positive family history, n/total count (%)	305	5/15 (33.3%)	150/290 (51.7%)	0.17
Presence of thyrotoxic symptoms, n/total count (%)	284	11/15(73.3%)	266/269 (98.9%)	<0.001
Presence of goiter, n/total count (%)	284	9/15 (60.0%)	186/269 (69.1%)	0.568
Presence of orbitopathy, n/total count (%)	284	0/15 (0%)	45/269 (16.7%)	0.14
Initial fT4 (ng/dL)	299	2.2 (2-2.8)	4.1 (3.1-5.2)	<0.001
Initial TSH (uIU/mL)	237	0.03 (0.03-0.1)	0.03 (0.03-0.04)	0.135
Initial TRAb (%)	290	27.2 (16.4-57.4)	58.8 (38.7-76.4)	0.004
Initial methimazole dose (mg/kg/day)	306	0.1 (0.1-0.2)	0.3 (0.2-0.4)	<0.001
Presence of adverse effects of methimazole, n/total count (%)	306	1/15 (6.7%)	25/291 (8.6%)	1

Data are presented as n/total count (%) for categorical variables and as medians (IQR) for continuous variables.

Significance level set at *p* < 0.05 (chi-square test or Fisher’s exact tests for categorical variables; Mann–Whitney *U* test for continuous variables). N/A, Not Applicable.

Clinical thyrotoxicosis was observed in 11 of 15 GD patients with T1D, which was significantly lower than in GD without T1D group (11/15 vs. 98.9%, *p* < 0.001). Among the GD patients with T1D, nine presented with goiter, and four had new-onset T1D concurrent with DKA and GD. Elevated HbA1c level was observed in two patients during hyperthyroidism, and normalized after euthyroidism was achieved. None of the patients in GD with T1D group exhibited Graves ophthalmopathy (GO) or other autoimmune disease at presentation. At diagnosis, the median levels of free T4 (2.2 vs. 4.1 ng/dL, *p* < 0.001) and TRAb (27.2% vs. 58.5%, *p* = 0.004) were significantly lower in GD with T1D group. However, the levels of TSH were similar between the two groups (0.03 vs. 0.03 µIU/mL, *p* = 0.135).

### Clinical course and outcome of GD patients with and without T1D

**Figure 1:**
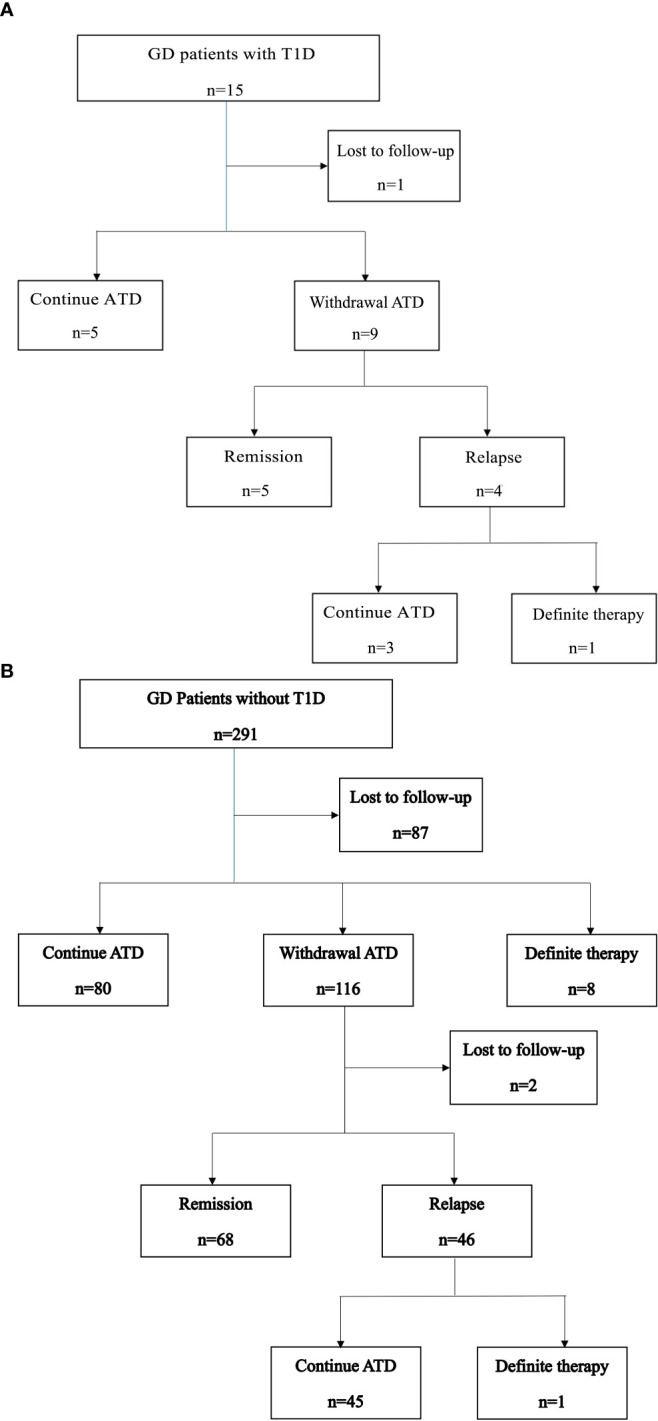
**(A)** Clinical course of GD patients with T1D. **(B)** Clinical course of GD patients without T1D.

Among the 15 GD patients with T1D, five continued ATD therapy, nine discontinued ATD, and one was lost to follow-up. Of the nine patients who discontinued ATD therapy, five achieved remission after a median of 2.7 years (IQR: 1.8–7.9 years) of treatment, while four experienced relapses (**Figure 1A**). Notably, one of the patients in the relapse group had maintained euthyroid for 5 years before relapse. One patient received RAI after relapse, which was unsuccessful. Subsequently, she underwent thyroidectomy six months after the RAI therapy.

The clinical course of GD patients without T1D is shown in **Figure 1B**. All the 291 patients received ATD as initial therapy. Among them, 80 (27.5%) continued ATD therapy for a median duration of 3.8 years. Eight patients (2.7%) underwent definitive therapy, including five who underwent surgery and three who received RAI, after a median of 3.8 years of ATD treatment. A total of 116 patients (39.9%) discontinued ATD therapy. Of these, 68 (23.4%) achieved remission, with a median time to remission of 5 years (IQR: 2.8–7.2 years). Forty-six patients (15.8%) experienced relapse, and one of them subsequently underwent surgery. Two patients were lost to follow-up after ATD withdrawal.

All patients received dose titration therapy. Adverse effects were uncommon, with one patient in GD with T1D group and 25 patients (8.6%) in the control group. Reported adverse effects included skin rash (N=18, 69.2%), liver function impairment (N=6, 23%), malaise (N=1, 3.8%), and group A streptococcal pharyngitis without documented agranulocytosis (N=1, 3.8%). No patients discontinued ATD due to adverse events.

**Figure 2:**
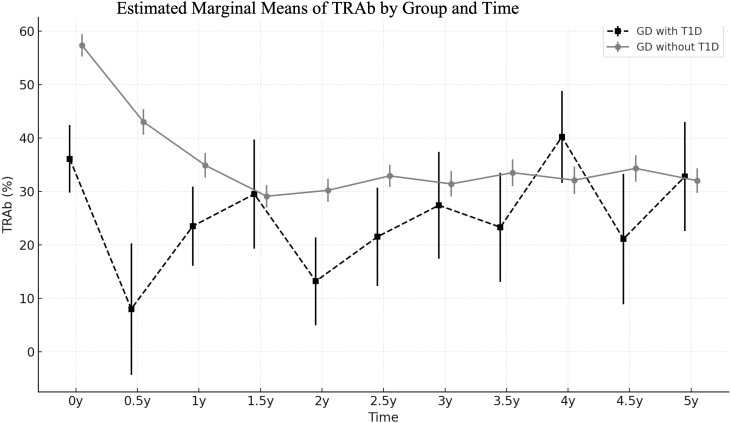
Trends of estimated marginal means of TRAb (%) in GD patients with and without T1D, analyzed using a linear mixed-effects model.

At diagnosis, GD patients with T1D were prescribed a significantly lower dose of methimazole compared to those without T1D (0.1 vs. 0.33 mg/kg/day, p < 0.001). Despite the lower dosage, the levels of free T4 normalized more rapidly in GD with T1D group (2.2 vs. 3.5 months, *p* = 0.031). TRAb levels were lower in the GD with T1D group at baseline and remained lower throughout follow-up, though the group effect did not reach significance (*p* = 0.062). Following treatment, the TRAb levels significantly declined over time (p < 0.001), with different trajectories observed between two groups (Group × Time interaction, p = 0.031). GD without T1D group showed a more rapid decline, whereas GD with T1D group exhibited a slower and fluctuating reduction over time. Pairwise comparisons further revealed significantly lower TRAb levels in the GD withT1D group at 0 and 0.5 years (p < 0.01), but the differences diminished after 1 year (**Figure 2**). The final outcomes including remission rate (5/15 vs. 23.4%, *p*= 0.377) and time to remission (2.7 vs. 5 years, *p*= 0.446) was similar between two groups (**Table 2**).

**Table 2:** Clinical outcome of pediatric GD patients with and without T1D.

Variable	N	GD with T1D N=15	GD without T1D N=291	P value
fT4 normalization time (months)	306	2.2 (1-4.2)	3.5 (1.7-7.5)	0.031
Remission, n/total count (%)	306	5/15 (33.3%)	68/291 (23.4%)	0.363
Male, n/total count (%)	43	1/5 (20%)	9/38 (23.6%)	1.000
Female, n/total count (%)	263	4/10 (40%)	59/253 (23.3%)	0.258
Time to remission (years)	73	2.7 (1.8-7.9)	5.0 (2.8-7.2)	0.446

Data are presented as n/total count (%) for categorical variables and as medians (IQR) for continuous variables.

Significance level set at *p* < 0.05 (Fisher’s exact test for categorical variables and Mann–Whitney *U* test for continuous variables).

## Discussion

In this retrospective study, we found that 2.3% of pediatric patients with T1D developed GD. Compared with GD patients without T1D, those with T1D exhibited fewer symptoms of thyrotoxicosis and had lower levels of free T4 and TRAb at GD diagnosis. These patients were also treated with lower doses of antithyroid drugs and required a shorter duration to achieve euthyroid status. Although GD patients with T1D were diagnosed at a relatively early stage of the disease, no significant differences were observed between the two groups either in remission rate or time to remission.

In our study, we observed that the prevalence of GD in pediatric patients with T1D was higher than that in Western countries. Few studies have explored the prevalence of hyperthyroidism in patients with T1D, particularly in Asian populations. Greco et al. (8) reported a GD prevalence of 3% in adults with T1D. In a study conducted in Italy, Lombardo et al. (16) reported a GD prevalence of 0.53% in young patients with diabetes, similar to that observed in the general Caucasian population. In a study involving a large diabetes database (Diabetes Prospective Follow-Up Registry), conducted in Austria and Germany, Dost et al. (9) reported that only 0.46% (276 out of 60,456) pediatric patients with T1D developed GD. Previous studies also reported a higher GD prevalence in Asian compared to Caucasian populations (17). Ethnic differences in HLA gene distribution may explain the higher prevalence of GD in East Asian populations. While HLA-DR3 is a major risk allele in Europeans, HLA-B*46, HLA-DRB1 Alleles are more strongly associated with GD in East Asians (18–20). These population-specific HLA variants likely influence autoimmune responses contributing to disease susceptibility and clinical variability (21).

In GD patients with T1D, the symptoms and signs of hyperthyroidism may be subtle or obscured by diabetic ketoacidosis (9). Four of our patients were simultaneously diagnosed with GD and T1D, presenting only with tachycardia or mild goiter. These findings underscore the importance of screening each newly diagnosed child or adolescent with T1D for thyroid autoimmunity, as recommended by the American Diabetes Association (22).

In our study, GD patients with T1D exhibited lower levels of free T4 and TRAb at diagnosis compared with those without T1D. Approximately one-third of our patients were diagnosed with GD before they developed clinical symptoms or signs. These patients’ free T4 levels normalized faster despite receiving lower dosages of methimazole. These findings are consistent with those of an Italian study, which reported mild clinical presentation and low free T4 and TRAb levels in the diabetic group at the time of GD diagnosis. Despite these findings, this Italian study did not provide details on methimazole dosages or treatment responses, specifically regarding the duration required for free T4 levels to normalize after treatment (16). Thyrotoxicosis affects glucose metabolism, impairs glucose utilization, and increases the risk of diabetic ketoacidosis and hypoglycemia in patients with T1D (13). A prospective follow-up registry conducted in Austria and Germany reported that T1D patients with comorbid hyperthyroidism were at a 2.3-fold higher risk of diabetic ketoacidosis and a 2-fold higher risk of severe hypoglycemia compared with T1D patients without hyperthyroidism (9). Thyroid dysfunction may exacerbate dyslipidemia by increasing the levels of total cholesterol, low-density lipoprotein, and triglyceride, thereby accelerating the progression of cardiovascular disease in patients with T1D (23). Therefore, early detection and treatment of GD may shorten the duration of thyrotoxicosis and mitigate the risk of acute and chronic complications associated with T1D.

Although early detection and treatment were associated with mild clinical presentation and low TRAb levels at GD diagnosis, we observed no significant differences in remission rate or time to remission between the two groups in our study. TRAb has been reported as a prognostic factor for GD, with high TRAb levels at diagnosis or upon cessation of therapy being associated with an increased risk of relapse (24, 25). In our study, we found that, despite lower TRAb levels at GD diagnosis, the decline in TRAb was slower and more variable over time in GD with T1D group. The intergroup difference in TRAb levels diminished by one year after treatment initiation, which may account for the comparable remission outcomes observed between the two groups.

## Strengths and limitations

This study has several limitations. First, the sample size was small in GD with T1D group. The disparity in sample size between the two groups reflects the low prevalence of T1D in the Asian population. As a result, the statistical comparisons should be interpreted with caution, as the small sample size in the GD with T1D group may limit the reliability and generalizability of the findings. Second, there was selection bias in patients in GD with T1D group. They were regularly checked-up, which explained the milder clinical presentation and lower dosage of MMI. Third, the lost to follow-up rate was relatively high in control group. In Taiwan, due to the high accessibility of medical centers and local clinics, patients are not restricted to a single healthcare provider. They may seek care directly at tertiary centers or switch healthcare facilities without requiring a referral. This flexibility contributes to high patient mobility and inconsistent follow-up, which may compromise the continuity and completeness of longitudinal data collection. Finally, patients with T1D were more likely to continue follow-up after remission of GD and may experience relapse several years later. Whereas patients in the control group may not return to our hospital years after remission, potentially leading to an underestimated relapse rate.

To the best of our knowledge, this is the first study to explore the clinical presentation and outcomes of GD in Asian children and adolescents with T1D. Compared with Caucasian populations, East Asian populations have a higher prevalence of GD, underscoring the importance of addressing GD in patients with T1D. Further prospective and multicenter studies with larger sample sizes are warranted.

## Conclusion

In our cohort, the prevalence of GD in pediatric patients with T1D was approximately 2.3%. Compared with GD patients without T1D, those with T1D exhibited lower levels of free T4 and TRAb at the time of GD diagnosis. Approximately one-third of GD patients with T1D presented without thyrotoxic symptoms or signs at the time of GD diagnosis. Early diagnosis and treatment of GD were associated with rapid normalization of free T4 levels, even with a lower dosage of methimazole. Although the remission rates did not significantly differ between two groups, annual thyroid function screening for patients with T1D is still recommended.

## Data Availability

The original contributions presented in the study are included in the article/supplementary material, further inquiries can be directed to the corresponding author/s.
